# Topological Path Planning in GPS Trajectory Data

**DOI:** 10.3390/s16122203

**Published:** 2016-12-21

**Authors:** Padraig Corcoran

**Affiliations:** School of Computer Science & Informatics, Cardiff University, Queen’s Buildings, 5 The Parade, Roath, Cardiff CF24 3AA, UK; corcoranp@cardiff.ac.uk; Tel.: +44-29-2087-4812

**Keywords:** path planning, GPS trajectory, topology

## Abstract

This paper proposes a novel solution to the problem of computing a set of topologically inequivalent paths between two points in a space given a set of samples drawn from that space. Specifically, these paths are homotopy inequivalent where homotopy is a topological equivalence relation. This is achieved by computing a basis for the group of homology inequivalent loops in the space. An additional distinct element is then computed where this element corresponds to a loop which passes through the points in question. The set of paths is subsequently obtained by taking the orbit of this element acted on by the group of homology inequivalent loops. Using a number of spaces, including a street network where the samples are GPS trajectories, the proposed method is demonstrated to accurately compute a set of homotopy inequivalent paths. The applications of this method include path and coverage planning.

## 1. Introduction

The task of path planning may be defined as computing a path or set of paths between two points in a given space. Path planning has many applications. If the space in question is a street network, path planning may act as a navigation aid for individuals. If the space in question is a robot configuration space, path planning may be used to transform a robot configuration or pose into a desired configuration or pose. Topological path planning is a type of path planning which does not distinguish between paths which are topological equivalent. For example, topological path planning in a space containing a single hole or obstacle would find at most two paths between any two locations in the space—specifically, one which traverses to the right and one which traverses to the left of the hole or obstacle. There exists many circumstances where it is most appropriate to perform topological path planning as opposed to any other type of path planning. For example, when the space in question is a street network, topological equivalent paths will correspond to traversing the same sequence of streets. If the purpose of path planning is to act as a navigation aid, it is not appropriate to distinguish between such paths.

From a computational perspective, path planning methods can broadly be divided into the two categories of combinatorial and sample-based methods. Combinatorial methods model the space using an exact but not necessarily accurate representation and subsequently search for paths within this representation (Chapter 6 of [1]). These methods return exact solutions, but, given the requirement for an exact representation of the space, they are not applicable in many cases. In addition, methods in this category generally do not scale efficiently with the dimension and number of obstacles in the space. Sample-baseds method overcome these challenges with the compromise of computing an approximate solution. Such methods represent the space using a set of samples drawn from the space and subsequently search for paths within this representation. This representation is an approximation, and, consequently, methods in this category return approximate solutions.

Despite the existence of a large number of methods for computing topologically inequivalent paths, these methods are combinatorial in nature and therefore exhibit the limitations described above [2]. The problem of computing topologically inequivalent paths using a sample-based approach has only recently been considered with [3] proposing the first such method. Specifically, the approach of [3] formulate the problem of computing topologically inequivalent paths in terms of doing inference with respect to co-homology, which is the dual of homology (*Homotopy* and *homology* are two fundamental path equivalence relations which are topological in nature (see Section 3)).

In this paper, we propose a novel sample-based topological path planning method that formulates the task in terms of doing inference with respect to homology. In particular, using techniques from the field of computational topology, it is possible to compute a canonical representative for each element in a generating set for the group of cycles which are homology inequivalent [4]. However, for the purposes of path planning, one is not interested in cycles but instead paths which begin and end at specified points. Overcoming this challenge represents the main contribution of this paper. To achieve this, we propose computing a canonical representative of a cycle which goes through the points in question. The orbit of this representative acted on by the cycles generated by the above generating set is then computed. This orbit corresponds to a set of canonical representatives for topologically inequivalent cycles which pass through the points in question. These cycles are subsequently transformed into homology inequivalent paths between the points in question. By considering the relationship which exists between homotopy and homology inequivalent paths, we demonstrate these paths to be also homotopy inequivalent. Relative to that proposed in [3], the method proposed in this paper represents a novel formulation of the problem—that is, in terms of doing inference with respect to homology as opposed to co-homology. The proposed method also exhibits superior computational complexity.

The layout of this paper is as follows: Section 2 reviews related works on topological path planning; Section 3 describes how the concept of topologically inequivalent paths may be defined formally in terms of homotopy and homology theory; and Section 4 describes the proposed sample-based topological path planning method. Finally, Section 5 and Section 6 present results and draw conclusions from this work, respectively.

## 2. Related Works

Topological path planning is a concept that exists in many research domains. In graph theory, there exist many algorithms, such as breadth-first search, for computing topologically inequivalent paths. How humans model the world is topological on some level and therefore topological path planning has been studied extensively in the field of spatial cognition [5,6]. Topological path planning has been demonstrated to have many applications in the robotics domain. For instance, it has been demonstrated that an effective strategy for exploring or performing coverage planning of a space is to identify the set of topologically inequivalent paths in that space and deploy a single robot along each [7]. Given the interest in topological path planning from all these domains, there exists a large spectrum of methods for performing this task. In this section, we review the most widely used combinatorial and sample-based methods.

Combinatorial methods for topological path planning generally operate as follows. They first convert the combinatorial representation of a space into a corresponding graph representation. Topologically inequivalent paths are subsequently determined by searching for paths in this graph which satisfy specific criteria. [8] defined the h-signature of a path which is a vector such that two paths which are topologically equivalent have the same h-signature. After converting the combinatorial representation of the space into a graph representation, topologically inequivalent paths are determined by searching for paths in this graph with different h-signatures. There exists many approaches for computing a graph representation such that all inequivalent paths in the graph correspond to topologically inequivalent paths in the original space. These graphs are commonly referred to as topological maps or graphs [9]. The most commonly used strategy for computing a topological graph is to compute the Generalized Voronoi Graph of the free space (GVG) which is related to the skeleton and forms a topological graph [10]. Another approach for computing a topological graph is described in [11] and employs a strategy where the space is decomposed into trapezoids that are used as platforms to construct the graph. A similar decomposition strategy based on mutual information is proposed by [12].

Sample-based methods for topological path planning have only recently been considered and are a consequence of recent advances in the domain of applied or computational topology [13]. In fact, the first such method was recently proposed by [3] where the authors formulate the problem in terms of doing inference with respect to co-homology, which is the dual of homology. Specifically, the authors construct a graph representation of the space and search for paths in this graph with different *signatures*. In contrast, the solution proposed in this paper formulates the problem in terms of doing inference with respect to homology. Relative to that proposed by [3], this solution is more intuitive in nature and has reduced computational complexity.

## 3. Homotopy and Homology Theory

*Homotopy* and *homology* are two fundamental equivalence relations which are topological in nature. In the following, we describe each of these relations in turn and the relationships that exist between them.

### 3.1. Homotopy Equivalence

Two paths are *homotopic* or *homotopy equivalent* if one can continuously deform into the other without intersecting any objects; to be continuously deformed is defined formally in [14]. The set of homotopy equivalence classes of paths in a topological space *X*, which begins and ends at a specified *base point* *p* in that space has a group structure known as the *fundamental group*, which is denoted $\pi_{1}\left(X,p\right)$. If the space in question is path connected, the fundamental groups corresponding to two distinct points in the space are isomorphic (see Theorem 7.13 of [14]). Therefore, the fundamental group of such a path connected space *X* is usually denoted $\pi_{1}\left(X\right)$, where the specification of a base point is omitted. Refs. [2,8,15] proposed methods for computing sets of homotopy inequivalent paths in two and three dimensions.

### 3.2. Homology Equivalence

Although homotopy equivalence classes are very appropriate in the context of path planning, they can be difficult to compute especially in higher dimensions [15]. For this reason, many researchers have begun to consider an alternative equivalence relation of homology [3,16]. Informally, two paths are *homology equivalent* or *homologous* if the region they enclose does not contain any holes. In the remainder of this subsection, we will formally define this concept and describe how we exploit the relationship between homotopy and homology equivalence toward computing a set of homotopy inequivalent paths.

Let K be an arbitrary simplicial complex such as a the Delaunay–Čech complex (see Appendix). A *k*-chain *c* is defined by Equation (1) where each $\sigma_{i}\in K$ is a *k*-simplex and each $\lambda_{i}$ is an element of $Z_{2}$ (integers modulo 2). Although we use $Z_{2}$ coefficients, as is common in path planning [3], coefficients in any field could be used:

(1) $$c=\sum \lambda_{i}\sigma_{i}.$$

The chain group $C_{k}\left(K\right)$ denotes the vector space over all *k*-chains. For a *k*-simplex $\sigma =[v_{1},\cdots ,v_{k+1}]$, the boundary map $\partial_{k}$ is defined by Equation (2), where $\hat{v_{i}}$ indicates that $v_{i}$ is deleted from the sequence. The boundary map is a map from a *k*-simplex to a sum of its (k−1)-simplex faces. For example, consider the 2-simplex $\left[v_{1},v_{2},v_{3}\right]$. The boundary map of this 2-simplex is the sum of 1-simplex faces $\left[v_{2},v_{3}\right]+\left[v_{1},v_{3}\right]+\left[v_{1},v_{2}\right]$. It can be easily proved that $\partial_{k+1}\partial_{k}=0:$

(2) $$\partial_{k}\sigma =\sum_{i=1}^{k+1}\left[v_{1},\cdots ,\hat{v_{i}},\cdots ,v_{k+1}\right].$$

The boundary map extends to the chain groups to form the chain complex $C_{*}$ as defined by Equation (3) [17]:

(3) $$\cdots \to C_{k+1}\left(K\right)\overset{\partial_{k+1}}{\to }C_{k}\left(K\right)\overset{\partial_{k}}{\to }C_{k-1}\left(K\right)\to \cdots .$$

**Figure 1:**
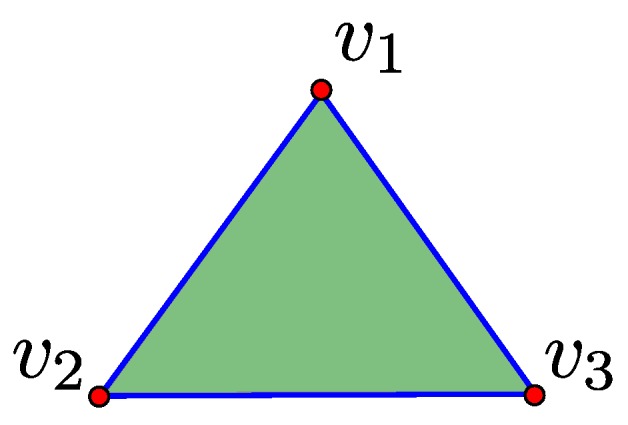
A simplicial complex K is illustrated where **red dots** represent 0-simplices, **blue lines** represent 1-simplices and **green triangles** represent 2-simplices.

A *k*-chain $c\in C_{k}\left(K\right)$ is called a *k*-boundary if there exists some $d\in C_{k+1}\left(K\right)$ such that c=∂d and a *k*-cycle if ∂c=0. To illustrate these conditions, consider the simplicial complex K of Figure 1. The 1-chain $\left[v_{2},v_{3}\right]+\left[v_{1},v_{3}\right]+\left[v_{1},v_{2}\right]\in C_{1}\left(K\right)$ is a 1-boundary because it equals the boundary map of the 2-chain $\left[v_{1},v_{2},v_{3}\right]\in C_{2}\left(K\right)$. Furthermore, the 1-chain $\left[v_{2},v_{3}\right]+\left[v_{1},v_{3}\right]+\left[v_{1},v_{2}\right]\in C_{1}\left(K\right)$ is a 1-cycle because applying the boundary map gives 0.

The set of *k*-boundaries and *k*-cycles are denoted by $B_{k}\left(K\right)$ and $Z_{k}\left(K\right)$, respectively. Both are subgroups of $C_{k}\left(K\right)$. As a consequence of $\partial_{k+1}\partial_{k}=0$, $B_{k}\left(K\right)\subseteq Z_{k}\left(K\right)$. The quotient group $H_{k}\left(K\right)=Z_{k}\left(K\right)/B_{k}\left(K\right)$ is called the *k*-homology group of K. The rank of $H_{k}\left(K\right)$ is called the kth Betti number and intuitively equals the number of *k*-dimensional holes in K. Using this intuition, the 0th Betti number corresponds to the number of connected components while the 1th Betti number corresponds to the number of one-dimensional holes. Since we are using $Z_{2}$ coefficients, a homology group with rank *n* has order $2^{n}$ [4] where the order corresponds to the number of homology classes.

The equivalence class of a *k*-cycle in $H_{k}\left(K\right)$ is denoted [c] and is called a *homology class*. Two *k*-cycles belonging to the same homology class are said to be *homologous*. For the purposes of path planning, we are interested in the homology classes of $H_{1}\left(K\right)$ because these correspond to trajectories in space.

Despite being a consequence of two distinct equivalence relations, the first homology and fundamental groups of a path-connected space have a simple relationship. Specifically, for a path connected space *X* containing a point *p*, there exists a surjective homomorphism from $\pi_{1}\left(X,p\right)$ to $H_{1}\left(K\right)$, whose kernel is the commutator subgroup of $\pi_{1}\left(X,p\right)$. That is, $H_{1}\left(K\right)$ is isomorphic to the Abelianization of $\pi_{1}\left(X,p\right)$. This homomorphism is known as the *Hurewicz homomorphism* [14,18]. In this paper, we exploit this relationship in the following manner. Our proposed path planning method finds a set of homology inequivalent paths. Since the Hurewicz homomorphism is surjective, these paths are also homotopy inequivalent.

## 4. Computing Homotopy Inequivalent Paths

**Figure 2:**
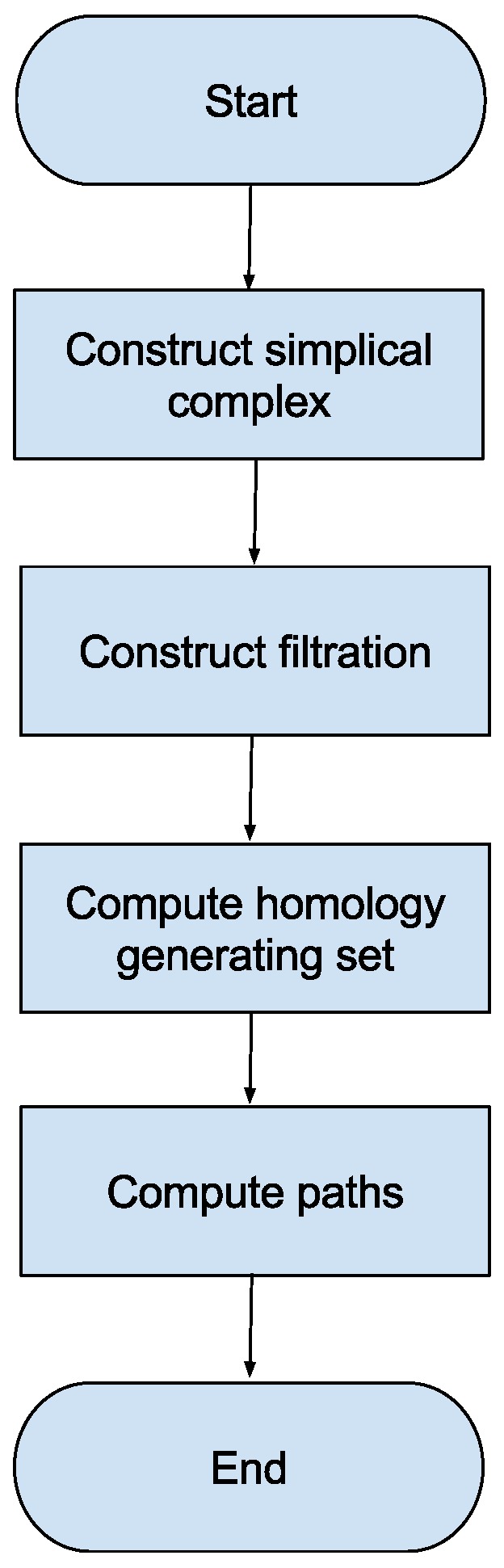
A flow chart of the proposed sample-based topological path planning method is displayed.

This section describes the proposed sample-based topological path planning method. This method contains a number of steps that are represented as a flow chart in Figure 2. The method first computes a simplicial complex K representation of the space in question using a set of samples from that space. This step is described in the Appendix. The method next constructs a representation of K known as a filtration which determines attributes of the simplices of K. A generating set which generates a canonical representative for each element in the homology classes of $H_{1}\left(K\right)$ is subsequently computed. Finally, this generating set is used to compute the desired set of paths. These steps are described in turn in the following subsections.

### 4.1. Filtration

**Figure 3:**
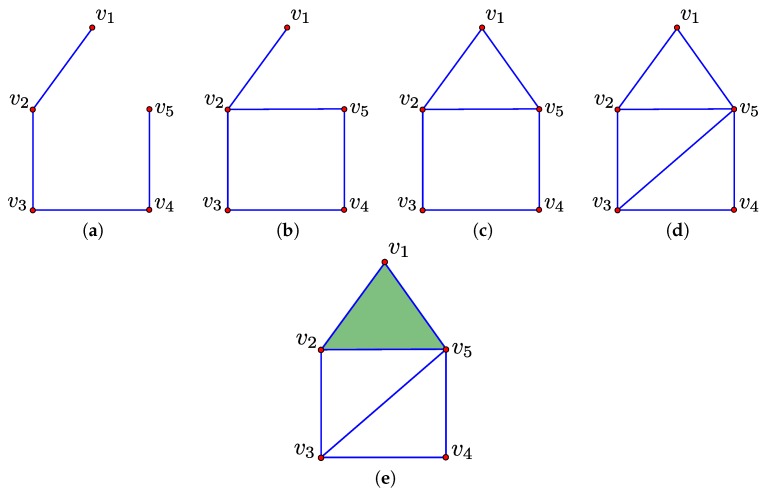
A subsequence of a filtration corresponding to a simplicial complex K is illustrated in (**a**–**e**) where **red dots** represent 0-simplices, **blue lines** represent 1-simplices and **green triangles** represent 2-simplices.

A sequence of simplicial complexes of the form defined in Equation (4) is called a filtration of K [4]. To illustrate this concept, consider the simplicial complex K represented in Figure 3e. A subsequence of a corresponding filtration is given by the sequence of simplicial complexes $K_{n-4}$, $K_{n-3}$, $K_{n-2}$, $K_{n-1}$ and $K_{n}$ represented in Figure 3a–e, respectively:

(4) $$\emptyset =K_{0}\subseteq K_{1}\subseteq \cdots \subseteq K_{n-1}\subseteq K_{n}=K.$$

In this work, we only consider filtrations where $K_{i-1}$ and $K_{i}$ differ by a single simplex which we denote $\sigma_{i}$. That is, $K_{i}$ is obtained by adding $\sigma_{i}$ to $K_{i-1}$. Let $d_{i}$ denote the dimension of $\sigma_{i}$. When adding $\sigma_{i}$ to $K_{i-1}$ to obtain $K_{i}$, two possible changes of the homology may occur [19]. Either an element in the $d_{i}$-homology group is created, in which case $\sigma_{i}$ is termed a *positive* simplex, or an element of the $\left(d_{i}-1\right)$-homology group is destroyed, in which case $\sigma_{i}$ is termed a *negative* simplex. To illustrate this, consider the filtration represented in Figure 3 and the action of adding the 1-simplex $\left[v_{1},v_{5}\right]$ to the simplicial complex represented in Figure 3b to obtain the simplicial complex represented in Figure 3c. This action creates an element in the 1-homology group, that is a 1-dimensional hole, and, therefore, $\left[v_{1},v_{5}\right]$ is a *positive* simplex. Now, consider the action of adding the 2-simplex $\left[v_{1},v_{2},v_{5}\right]$ to the simplicial complex represented in Figure 3d to obtain the simplicial complex represented in Figure 3e. This action destroys an element in the 1-homology group, and, therefore, $\left[v_{1},v_{2},v_{5}\right]$ is a *negative* simplex.

For each negative simplex $\sigma_{j}$, we associate a positive simplex $\sigma_{i}$ where i<j such that $\sigma_{j}$ destroys the element of the homology group created by $\sigma_{i}$. The pair $\left(\sigma_{i},\sigma_{j}\right)$ is called a *persistence pair* and the value j−i its *index persistence*. In the context of the filtration represented in Figure 3, $\left(\left[v_{1},v_{5}\right],\left[v_{1},v_{2},v_{5}\right]\right)$ is a persistence pair because adding $\left[v_{1},v_{2},v_{5}\right]$ destroys the element in the 1-homology group which was created when $\left[v_{1},v_{5}\right]$ was added. The index persistence in this case is n−(n−2)=2.

A subset of positive simplices, which create an element of the homology group that is subsequently not destroyed, is not paired. These are referred to as *essential simplices* [19]. In the context of the filtration represented in Figure 3, the 1-simplex $\left[v_{2},v_{5}\right]$ is an essential simplex. Every simplex of K belongs either to a persistence pair or is an essential simplex. For the purposes of this work, we are primarily interested in essential 1-simplices because these create the elements of the 1-homology group $H_{1}\left(K\right)$. The number of essential k-simplices is equal to the kth Betti number.

In this work, given a simplicial complex K we construct a corresponding filtration by ordering the simplices in K such that simplices of lower dimension precede simplices of higher dimension and simplices of the same dimension are ordered arbitrarily. We then define $K_{i}$ to be that simplicial complex which is obtained by adding the *i*th simplex $\sigma_{i}$ in this ordering to $K_{i-1}$. Since simplices are ordered by dimension, this corresponds to a valid filtration [4]. There exists a number of algorithms for computing the persistence pairs and essential simplices for a given simplicial complex K [19,20,21]. In this work, we employ the algorithm of [21].

### 4.2. Computing Paths

**Figure 4:**
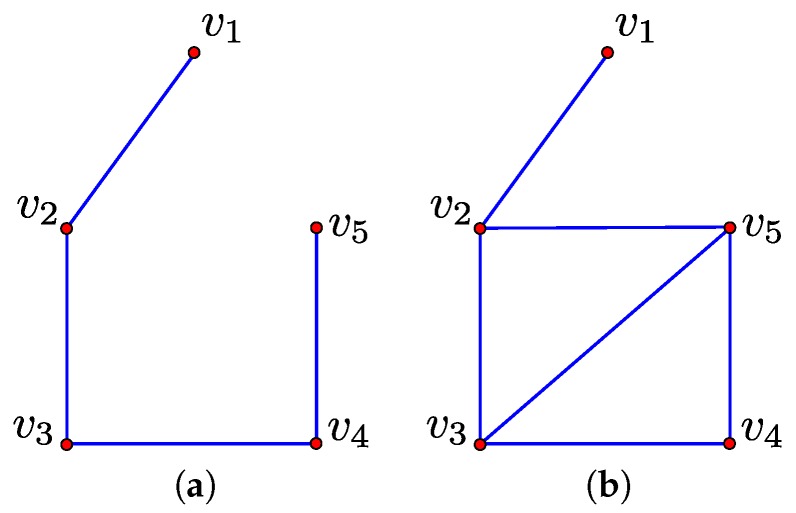
The set of negative 1-simplices and the set of negative and essential 1-simplices in the filtration of Figure 3 are displayed in (**a**,**b**), respectively.

The set of negative 1-simplices in the filtration of K forms a spanning tree of the 0-simplices in K [22,23]. This property is illustrated by Figure 4a which displays the set of negative 1-simplices corresponding to the filtration of Figure 3. When considering homology with $Z_{2}$ coefficients, as we do in this paper, the set of negative and essential 1-simplices in K form a graph with the following property [22,23]. Each simple cycle that contains a single essential 1-simplex corresponds to an element in a generating set for canonical representatives of elements in the homology classes of $H_{1}\left(K\right)$. We denote this generating set as $G_{K}$. The set generated by $G_{K}$, denoted $\langle G_{K}\rangle$, corresponds to canonical representatives for all elements in the homology classes of $H_{1}\left(K\right)$. To illustrate these concepts, consider again the filtration of Figure 3; this filtration has two essential 1-simplices of $\left[v_{3},v_{5}\right]$ and $\left[v_{3},v_{5}\right]$, and, in turn, two elements in the corresponding generating set $G_{K}$. These elements are the cycles $\left[v_{2},v_{3}\right]+\left[v_{2},v_{5}\right]+\left[v_{3},v_{4}\right]+\left[v_{4},v_{5}\right]$ and $\left[v_{3},v_{4}\right]+\left[v_{3},v_{5}\right]+\left[v_{4},v_{5}\right]$. The group operator in the case of $Z_{2}$ coefficients corresponds to symmetric difference or exclusive OR. For example, applying the group operator to the above elements $\left[v_{2},v_{3}\right]+\left[v_{2},v_{5}\right]+\left[v_{3},v_{4}\right]+\left[v_{4},v_{5}\right]$ and $\left[v_{3},v_{4}\right]+\left[v_{3},v_{5}\right]+\left[v_{4},v_{5}\right]$ gives the element $\left[v_{2},v_{3}\right]+\left[v_{2},v_{5}\right]+\left[v_{3},v_{5}\right]$.

**Figure 5:**
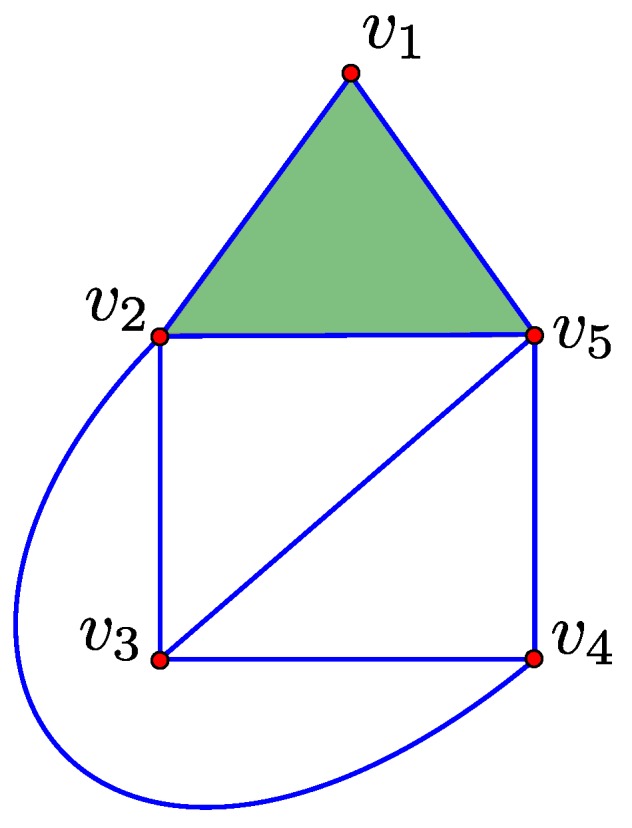
The 1-simplex [p,q] is added to the simplicial complex of Figure 3.

The canonical representatives of elements in the homology classes of $H_{1}\left(K\right)$ correspond to cycles in the space. However, for the purposes of path planning, one is not interested in cycles but paths in the space which begin and end at specified points. We overcome this challenge as follows. Let *p* and *q* be distinct 0-simplices in the simplicial complex K for which we wish to compute a set of homotopy inequivalent paths between. Let [p,q] be an additional 1-simplex which is added to K to obtain a new simplicial complex $K_{pq}$. To illustrate this construction, consider again the filtration of Figure 3 and the case where *p* and *q* correspond to the 0-simplices, $v_{2}$ and $v_{4}$, respectively. In this case, $K_{pq}$ corresponds to that simplicial complex which is represented in Figure 5. A filtration of $K_{pq}$ is defined in a similar manner to that of K, except that [p,q] is the last simplex added.

It is evident that [p,q] is a positive 1-simplex in the filtration of $K_{pq}$ and the canonical representative that it corresponds to is a cycle which passes through *p* and *q*. We denote this canonical representative as $g_{pq}$. Therefore, the orbit of $g_{pq}$ acted on by $\langle G_{K}\rangle$, which is denoted $\langle G_{K}\rangle \left(g_{pq}\right)$ and defined in Equation (5) [24], corresponds to a set of canonical representatives for homology inequivalent cycles which pass through *p* and *q*:

(5) $$\langle G_{K}\rangle \left(g_{pq}\right)=\left\{g_{pq}g:g\in \langle G_{K}\rangle \right\}.$$

To illustrate this, consider again the simplicial complex $K_{pq}$ represented in Figure 5, where *p* and *q* correspond to the 0-simplices $v_{2}$ and $v_{4}$, respectively. The canonical representative $g_{pq}$ corresponds to the cycle $\left[v_{2},v_{3}\right]+\left[v_{2},v_{4}\right]+\left[v_{3},v_{4}\right]$. The orbit of $g_{pq}$ acted on by $\langle G_{K}\rangle$ contains the cycles $\left[v_{2},v_{3}\right]+\left[v_{2},v_{4}\right]+\left[v_{3},v_{4}\right]$, $\left[v_{2},v_{3}\right]+\left[v_{2},v_{4}\right]+\left[v_{3},v_{5}\right]+\left[v_{4},v_{5}\right]$, $\left[v_{2},v_{4}\right]+\left[v_{2},v_{5}\right]+\left[v_{3},v_{4}\right]+\left[v_{3},v_{5}\right]$ and $\left[v_{2},v_{4}\right]+\left[v_{2},v_{5}\right]+\left[v_{4},v_{5}\right]$. These cycles are homology inequivalent and all pass through *p* and *q*.

The set of cycles in the orbit of $g_{pq}$ acted on by $\langle G_{K}\rangle$ can be transformed into a set of homology inequivalent paths between *p* and *q* by removing the 1-simplex [p,q] from each. For example, removing the 1-simplex $\left[v_{2},v_{4}\right]$ from each of the above cycles gives a set of homology inequivalent paths between $v_{2}$ and $v_{4}$. Specifically, these are the paths $\left[v_{2},v_{3}\right]+\left[v_{3},v_{4}\right]$, $\left[v_{2},v_{3}\right]+\left[v_{3},v_{5}\right]+\left[v_{4},v_{5}\right]$, $\left[v_{2},v_{5}\right]+\left[v_{3},v_{4}\right]+\left[v_{3},v_{5}\right]$ and $\left[v_{2},v_{5}\right]+\left[v_{4},v_{5}\right]$. By the Hurewicz homomorphism, all homology inequivalent paths between *p* and *q* created using the above approach are also homotopy inequivalent.

### 4.3. Computational Complexity

In this section, we examine the computational complexity of the proposed path planning method. We examine each of the steps in this method in turn. Computing the simplicial complex varies in complexity depending on the type of simplicial complex used. In this work, a Delaunay–Čech complex was used which requires exponential time to compute [25]. If computational complexity is a concern, one could use an alternative simplicial complex, such as the Vietoris–Rips complex which can be computed in linear space and time [26,27]. Using the method of [19], the set of negative and essential 1-simplices can be computed in polynomial time and space complexity. For a given essential 1-simplex, we compute the corresponding element in the generating set for canonical representatives as follows. We construct the spanning tree of negative 1-simplices. We next compute the single simple path which exists between the two 0-simplices contained in the boundary of the essential 1-simplex in question. The canonical representative is subsequently obtained by adding the essential 1-simplex to the set of 1-simplices in this path. This can be computed in time and space complexity that is linear in the number of samples using a breadth first search. This process is repeated for each essential 1-simplex, and, therefore, the overall time and space complexity for computing the elements in the generating set is polynomial. Computing the paths in question by computing the orbit is achieved in time that is linear in the number of paths.

The proposed method exhibits superior computational complexity to that proposed by [3]. Having computed a simplical complex, the method of [3] subsequently requires searching for paths in a graph where the size of the graph grows exponentially in the number of holes in the space. Consequently, as noted by the authors, these graphs quickly grow too large to fit into memory. On the other hand, having computed a simplical complex, the method proposed in this paper can compute paths in polynomial time.

## 5. Results

The proposed path planning method has a single parameter *r* corresponding to the radius of the Delaunay–Čech complex (see Equation (A4)). A commonly employed strategy for automatically setting this parameter is through the application of persistent homology [16,28]. This approach basically considers all values of *r* in a specified interval and only regards those elements of $H_{1}\left(K\right)$ which exist for a sufficiently large range within this interval to be significant. For all results presented in this paper, the parameter *r* was set manually; however, persistent homology could be employed to automate this task if necessary.

Towards demonstrating the ability of the proposed method to discover homotopy inequivalent paths, a number of simulated and real spaces were considered. We consider each of these in the following two subsections. A comparison to the method proposed by [3] is accomplished by comparing results achieved in homotopy equivalent spaces. It is demonstrated that both methods produce the same set of paths up to homotopy equivalence with the proposed method computing these in a more efficient manner.

### 5.1. Simulated Spaces

**Figure 6:**
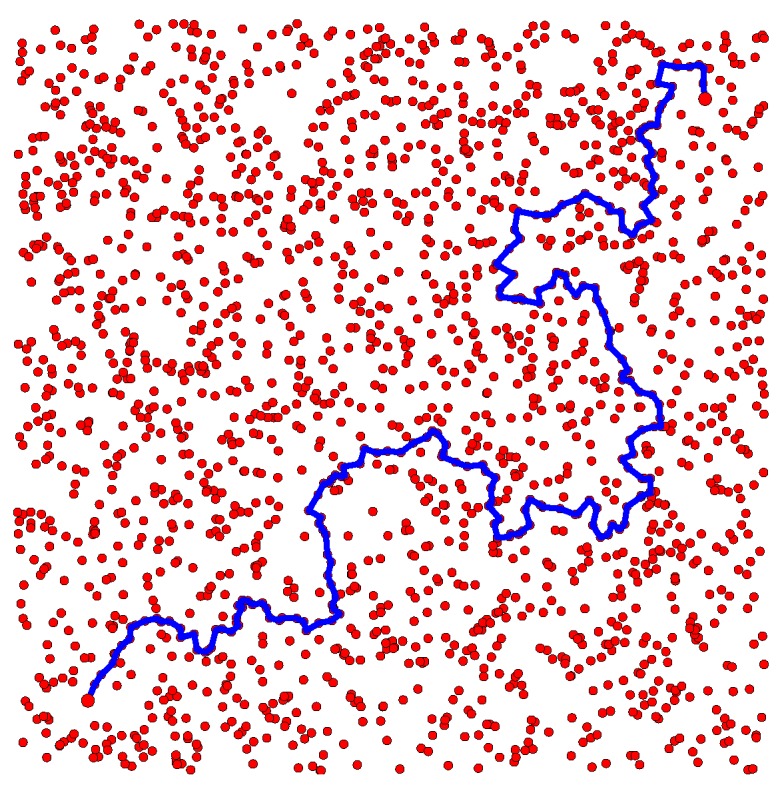
Two thousand samples are represented by **red dots**. For two points located in the lower left and top right of the space, the single path returned by the proposed path planning method is represented by a **blue line**.

Figure 6 displays two thousand points uniformly sampled from a simulated space that is a subset of $R^{2}$. The 0th and 1th Betti numbers of this space are both zero. For two points located in the bottom left and top right of this space, the proposed path planning method computed a set of homotopy inequivalent paths that contains a single element. The path in question is illustrated in Figure 6. Given that there are no holes in this space, there exists at most one homotopy inequivalent path between any two points. As such, the set of paths returned by the proposed method accurately models the set of homotopy inequivalent paths in the space.

**Figure 7:**
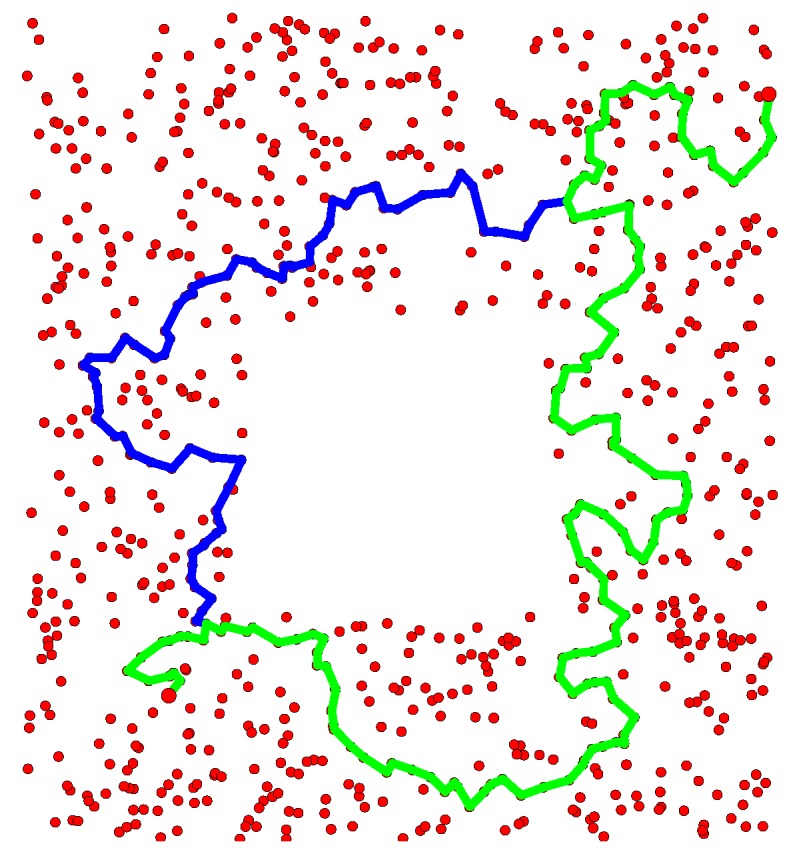
Two thousand samples are represented by **red dots**. For two points located in the lower left and top right of the space, the two paths returned by the proposed path planning method are represented by **blue** and **green lines**.

Figure 7 displays two thousand points uniformly sampled from a simulated space that is also a subset of $R^{2}$. The 0th and 1th Betti numbers of this space are zero and one, respectively. For two points located in the bottom left and top right of this space, the proposed path planning method computed a set of homotopy inequivalent paths that contains two elements. The paths in question are illustrated in Figure 6. This space is homotopy equivalent to those spaces represented in Figures 1 and 8 of [3], where paths are computed between a similar pair of points. By comparing the results obtained by both methods, it is evident that they return the same set of homotopy inequivalent paths.

**Figure 8:**
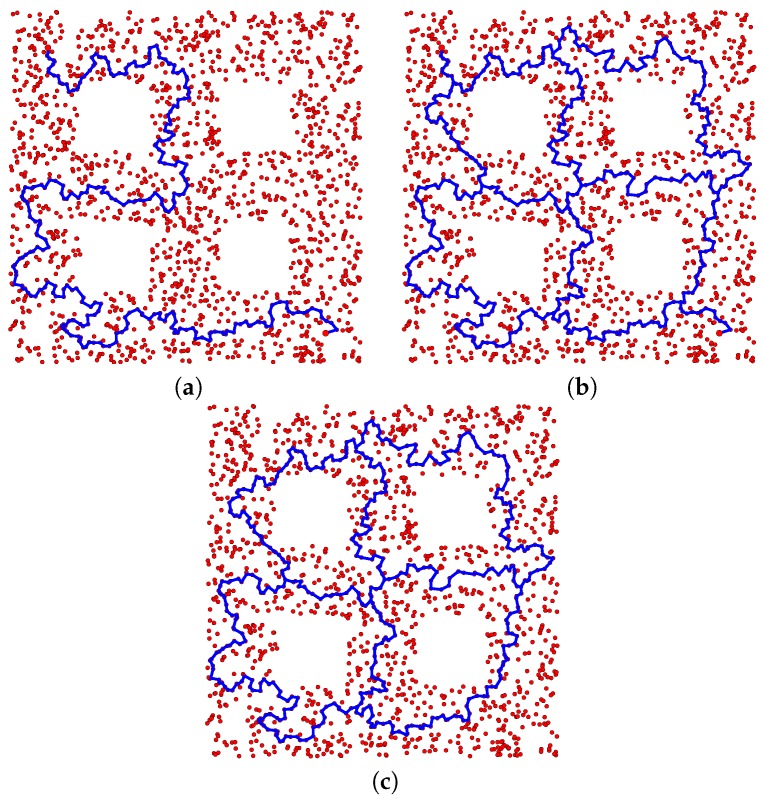
Two thousand samples are represented by **red dots** in (**a**). For two points located in the top left and bottom right of the space, a single path returned by the proposed path planning method is represented by a **blue line** in (**a**). All paths returned by the proposed method are represented by **blue lines** in (**b**). The elements of $\langle G_{K}\rangle$ are represented in (**c**).

Figure 8a displays two thousand points uniformly sampled from a simulated space that is a subset of $R^{2}$. The 0th and 1th Betti numbers of this space are zero and four, respectively. For two points located in the top left and bottom right of this space, the proposed path planning method returned a set of homotopy inequivalent paths containing twelve elements. A single path in this set is represented in Figure 8a, while all elements in the set are represented in Figure 8b. Figure 8c displays the elements of $\langle G_{K}\rangle$. Through a comparison of Figure 8b,c, it is evident that the proposed method effectively transforms the cycles in $\langle G_{K}\rangle$ into a corresponding set of suitable paths. As such, these paths effectively explore the space.

**Figure 9:**
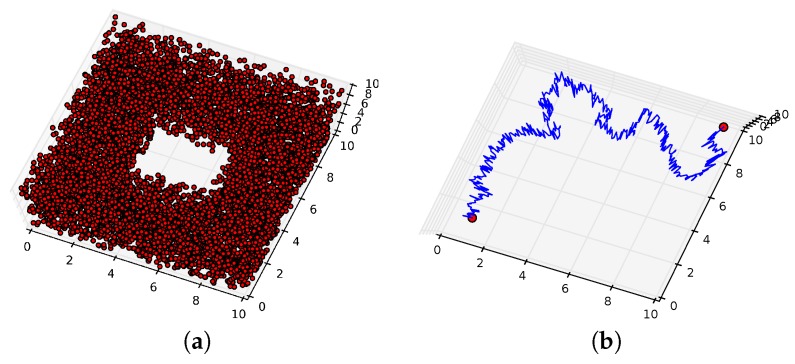
Ten thousand points sampled from a space are represented by **red dots** in (**a**). For two points located in the left and right of the space, a single path is represented by a **blue line** in (**b**).

The proposed path planning method can be applied to spaces of high dimension. To illustrate this, the following simulated spaces were considered. Figure 9a displays ten thousand points uniformly sampled from a simulated space which is a subset of $R^{3}$. The 0th, 1th and 2th Betti numbers of this space are one, one and zero respectively; this is, it is homeomorphic to a solid torus. For two points located in the left and right of this space, the proposed path planning method returned a set of homotopy inequivalent paths containing two elements. Figure 9b displays one of these paths which traverses a path above the hole in the space. The other path in the set traverses a path below the hole in the space.

**Figure 10:**
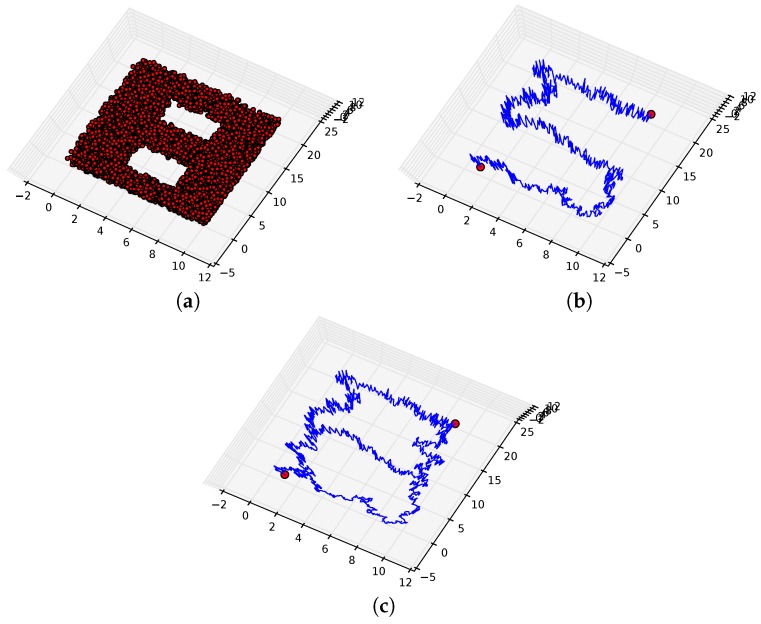
Two thousand samples are represented by **red dots** in (**a**). For two points located in the left and right of the space, a single path returned is represented by a **blue line** in (**b**). All paths returned are represented by **blue lines** in (**c**).

Figure 10a displays ten thousand points uniformly sampled from a simulated space that is a subset of $R^{3}$. The 0th, 1th and 2th Betti numbers of this space are one, two and zero, respectively; that is, it is homeomorphic to a solid two-holed torus. For two points located in the left and right of this space, the proposed path planning returned a set of homotopy inequivalent paths containing four elements. Figure 10b displays one of the paths in this set while all paths in the set are represented in Figure 10c. It is evident that the set of all paths surround each hole in the space, and, as such, these paths effectively explore the space.

### 5.2. Real Spaces

To demonstrate the proposed path planning method on a real space, we considered the street network for the city for Chicago which is a subset of $R^{2}$. The samples in question correspond to a publicly available dataset of GPS trajectories captured by 13 university shuttle buses serving the University of Illinois at Chicago campus [29] (see Section 9.1; a website proving links to the data can be found here (https://www.cs.uic.edu/bin/view/Bits/Software)).

**Figure 11:**
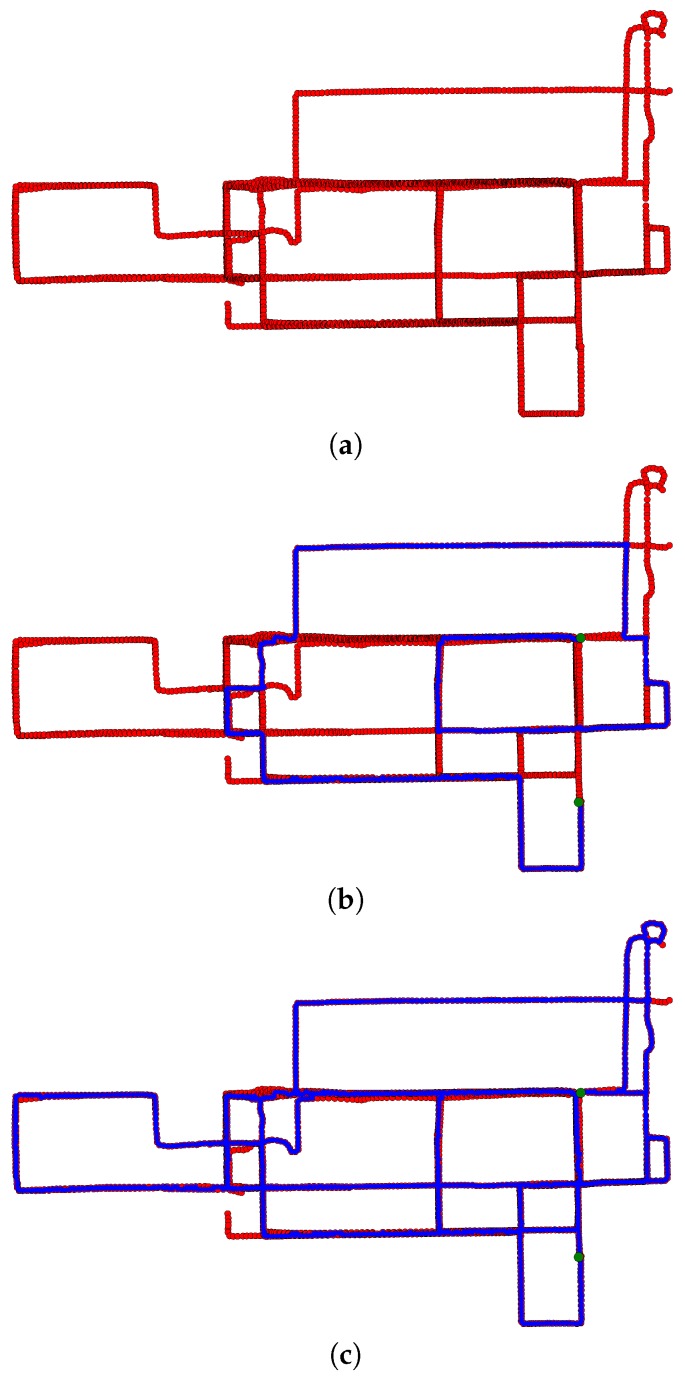
A total of 4432 GPS points corresponding to 50 trajectories captures by vehicles traversing the Chicago street network are displayed in (**a**). For two points represented by **green dots** and located just to the right of center, a single path returned by the proposed path planning method is represented by a **blue line** in (**b**). All paths returned by the proposed method are represented by **blue lines** in (**c**).

The area in question contains a mixture of low-, mid- and high-rise buildings. As a consequence, the GPS positional accuracy was error prone with some traces showing consistent errors over 100 m. From this dataset, 50 individual trajectories, containing 4432 individual GPS points, were considered. Figure 11a displays the trajectories in question. For two points located in the right of this space, a set of homotopy inequivalent paths were computed using the proposed method. Figure 11b displays one of the paths in this set while all paths in the set are displayed in Figure 11c. Again, it is evident that the set of all paths effectively explore the space.

## 6. Conclusions

The paper proposes a novel sample-based path planning method for computing a set of homotopy inequivalent paths between two points in a space. The fundamental contribution of this paper is the insight that such a set of paths may be obtained by computing the orbit of a cycle passing through the points in question acted on by the group of homology inequivalent loops. Results obtained on simulated and real data demonstrate the utility of this method.

The paths computed using the proposed path planning method are not localized optimally. For example, the path displayed in Figure 6 is not the shortest or smoothest path between the points in question. As discussed by [30], computing localized paths using homology is challenging because homology is a topological concept that does not consider such non-topological properties. One potential solution to overcoming this challenge would be to subsequently optimize the localizing of each computed path in a manner similar to that proposed by [31]. The authors hope to implement such a solution in future work.

## Figures and Tables

**Figure A1:**
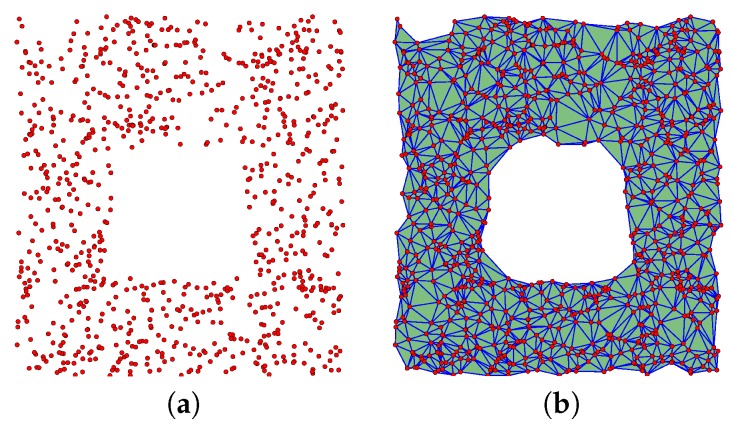
A set of samples from a simulated space are displayed in (**a**). The corresponding Delaunay–Čech complex for a given value of *r* is displayed in (**b**) where **red dots** represent 0-simplices, **blue lines** represent 1-simplices and **green triangles** represent 2-simplices.

## Appendix A

In this section, we describe how a space may be reconstructed from a set of samples using a combinatorial representation known as a simplical complex. Let $X=\{x_{1},\cdots ,x_{m}\}$ be a set of samples from a space that is a subset of $R^{n}$. Let ∥.∥ denote the Euclidean norm and $B_{r}\left(x\right)=\{y\in R^{n}|∥x-y∥<r\}$ for r≥0 denote a closed ball of radius *r* centred at *x*. The *r*-neighbourhood $X_{r}$, as defined by Equation (A1), represents an intuitive means of reconstructing the space. However, this is an abstract mathematical representation upon which computation is difficult. One solution to overcoming this challenge is to reconstruct the space using a combinatorial representation known as a simplicial complex upon which computations may be performed:

(A1) $$X_{r}=\overset{n}{\underset{i=1}{⋃}}B_{r}\left(x_{i}\right).$$

A simplicial complex K is a family of finite subsets of a universal set such that, for each *σ* in K, all subsets of *σ* are also in K. A set *σ* in K is called a *k*-simplex if $\left|\sigma \right|=k+1$, where $\left|.\right|$ represents the cardinality of the set in question. The faces of a simplex *σ* correspond to all simplices *τ* where τ⊂σ [13,32].

There exists a number of different simplicial complexes that may be used to reconstruct a space. In this paper, we consider the Delaunay–Čech complex, which has the attractive property that it is homotopy equivalent to $X_{r}$, where homotopy equivalence is an equivalence relation on topological spaces (see Chapter 7 in [14]). The Delaunay–Čech complex is equal to the intersection of a Čech complex and Delaunay triangulation, which we now describe in turn.

The Čech complex $C_{r}\left(X\right)$ for r≥0, as defined in Equation (A2), is the abstract simplicial complex whose *k*-simplices correspond to unordered (k+1)-tuples of points ${\left\{x_{\alpha }\right\}}_{0}^{k}$ whose closed r/2 neighbourhoods intersect [32]. The Čech complex $C_{r}\left(X\right)$ is homotopy equivalent to $X_{r}$:

(A2) $$C_{r}\left(X\right)=\left\{\sigma \subseteq X:\underset{x\in \sigma }{⋂}B_{r}\left(x\right)\neq \emptyset \right\}.$$

Let $V_{x}$ denote the Voronoi cell containing *x* [4]. Consider the simplicial complex D(X), as defined by Equation (A3), which corresponds to the Delaunay triangulation of *X*. This simplicial complex has the attractive property that the maximum dimension of any simplex it contains is *n* the dimension of the space. However, it does not have a scale parameter, and, therefore, it cannot accurately reconstruct spaces containing holes. This issue may be addressed by considering the Delaunay–Čech complex $DC_{r}\left(X\right)$ for r≥0, which is defined by Equation (A4) and contains those simplices which lie at the intersection of D(x) and $C_{r}\left(X\right)$. The Delaunay–Čech complex is homotopy equivalent to $B_{r}\left(x\right)$ [25]. To illustrate the Delaunay–Čech complex, consider Figure A1a, which displays a set of samples from a simulated space. The corresponding Delaunay–Čech complex for a given value of *r* is displayed in Figure A1b:

(A3) $$D\left(X\right)=\left\{\sigma \subseteq X:\underset{x\in \sigma }{⋂}V_{x}\neq \emptyset \right\},$$

(A4) $$DC_{r}\left(X\right)=D\left(X\right)\cap C_{r}\left(X\right).$$
